# The Significance of Aldehyde Dehydrogenase 1 in Cancers

**DOI:** 10.3390/ijms26010251

**Published:** 2024-12-30

**Authors:** Anh L. Nguyen, Caroline O. B. Facey, Bruce M. Boman

**Affiliations:** 1 Department of Biological Sciences, University of Delaware, Newark, DE 19716, USA; nguyena@udel.edu; 2 Center for Translational Cancer Research, Helen F. Graham Cancer Center and Research Institute, 4701 Ogletown-Stanton Road, Newark, DE 19713, USA; caroline.facey@christianacare.org; 3 Department of Pharmacology and Experimental Therapeutics, Thomas Jefferson University, Philadelphia, PA 19107, USA

**Keywords:** aldehyde dehydrogenase, ALDH isoenzymes, retinoic acid, cancer stem cells, colorectal cancer, breast cancer, lung cancer, gastric cancer, cervical cancer, melanoma, prostate cancer, renal cancer

## Abstract

The goal of this paper is to discuss the role of ALDH isozymes in different cancers, review advances in ALDH1-targeting cancer therapies, and explore a mechanism that explains how ALDH expression becomes elevated during cancer development. ALDH is often overexpressed in cancer, and each isoform has a unique expression pattern and a distinct role in different cancers. The abnormal expression of ALDHs in different cancer types (breast, colorectal, lung, gastric, cervical, melanoma, prostate, and renal) is presented and correlated with patient prognosis. ALDH plays a significant role in various cellular functions, such as metabolism, oxidative stress response, detoxification, and cellular differentiation. Among the ALDH families, ALDH1 has gained considerable attention as a cancer stem cell (CSC) marker due to its significant role in the maintenance of stemness and the differentiation of stem cells (SCs), along with its involvement in tumorigenesis. A description of the cellular mechanisms and physiology of ALDH1 that underlies cancer development is provided. Moreover, current advances in ALDH1-targeting cancer therapies are discussed.

## 1. Background Information

The aim of this background is to discuss the function and superfamily structure of aldehyde dehydrogenase (ALDH) proteins in relation to cancer development, growth, and treatment. The ALDH group of genes functions as enzymes that catalyze the oxidation of endogenous and exogenous aldehyde substrates to carboxylic acids in certain life processes. In this paper, we start with a description of how ALDH function might be crucial to the development of cancer. We then provide a description of ALDH superfamilies, isoenzymes, protein structure, and cellular physiology. Finally, a deliberation on the role that ALDH1 plays in the development of breast, colorectal, lung, gastric, and other cancers is delivered.

**Figure 1:**
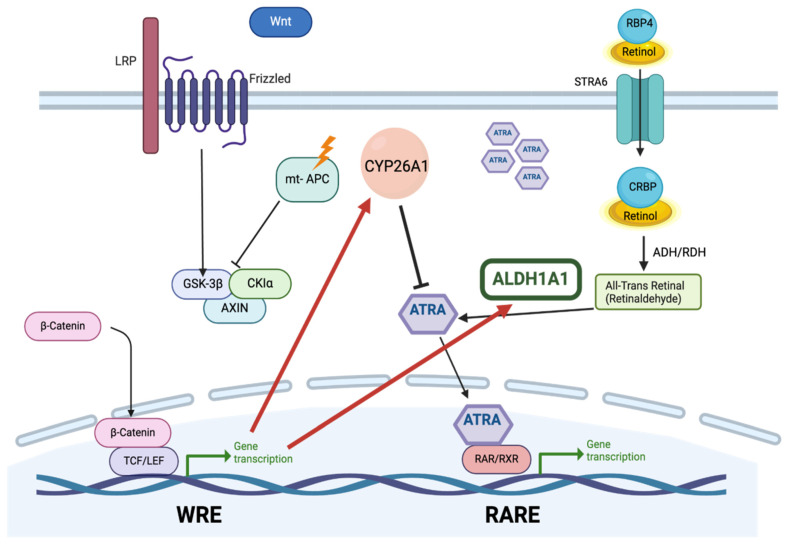
Proposed feedback mechanism of WNT signaling and RA pathway. APC cancer-driver mutations that constitutively activate WNT signaling by preventing the formation of GSK-3β/CKIɑ/AXIN/APC complex, hence β-catenin is able to access the DNA and bind to the TCF/LEF transcription factor to promote transcription of CYP26A1 transcription—a metabolizing enzyme of RA. Retinol is transported to the cell by RBP4 and then converted to all-trans retinal and then to all-trans retinoic acid (ATRA).

ALDH1A proteins play a key role in many biological processes, such as development, homeostasis, and tumorigenesis, through their enzymatic activity in retinoic acid (RA) signaling by converting retinal to RA (Figure 1). This ALDH-based step in the RA pathway is important because RA signaling controls cellular differentiation. Indeed, we found that retinoid agents, such as all-trans RA (ATRA), can induce differentiation of ALDH-positive stem cells (ALDH+ SCs) along the neuroendocrine cell (NEC) lineage [1,2]. Additionally, our studies reveal that RA receptors are selectively expressed in ALDH+ SCs [1], which indicates RA signaling mainly occurs in ALDH+ SCs. Because ALDH activity is increased in normal and malignant stem cells (SCs), technologies such as the ALDEFLUOR assay have been developed to utilize ALDH enzymatic activity as a marker for quantifying and isolating SC populations. Indeed, we and others have shown that ALDH+ SCs become overpopulated in the development of colorectal cancer (CRC) [3] and other cancers [4]. Our study of why ALDH is selectively expressed in SCs addresses a gap-in-our-knowledge. It may seem counterintuitive that ALDH, which is a key component in the RA pathway and RA signaling induces cellular differentiation, is also selectively expressed in SCs that are undifferentiated. One explanation is that the reason that ALDH is expressed in SCs is because it gives them their potential for multilineage differentiation. It is also perplexing why increased ALDH expression and overpopulation of ALDH+ SCs occur in cancers. An explanation could come from the finding that RA metabolizing enzyme CYP26A1, which metabolizes RA, decreases RA signaling, and impedes cell differentiation, is also overexpressed in CRC cells [5] and other cancer types [6,7,8]. Thus, the hypothesis is developed that an increase in ALDH expression occurs upstream in the RA pathway through a feedback mechanism in the cell’s response to compensate for low intracellular RA levels caused by increased CYP26A1 (Figure 1). This mechanism may explain how *Adenomatous Polyposis Coli (APC)* cancer-driver mutations that constitutively activate WNT signaling and increase CYP26A1 expression led to SC overpopulation and incomplete differentiation during tumor development.

Upregulation of CYP26A decreases ATRA expression. In response to low intracellular ATRA levels, ALDH1A1 is proposed to be upregulated by both WNT and RA signaling in an attempt to increase the intracellular ATRA level. ALD = alcohol dehydrogenase; ALDH = aldehyde dehydrogenase; CRBP = Cellular Retinol-Binding Protein, LEF = Lymphoid enhancer factor; WRE = WNT regulatory element; RARE = retinoic acid regulatory element; RBP4 = Retinol Binding Protein 4; RDH = retinol dehydrogenase; TCF = T Cell Factor; WRE = WNT regulatory element.

### 1.1. ALDH Superfamilies and Their Function

To date, the human ALDH superfamily of genes is comprised of 11 major families: ALDH1, ALDH2, ALDH3, ALDH4, ALDH5, ALDH6, ALDH7, ALDH8, ALDH9, ALDH16, and ALDH18, and four subfamilies [9,10]. In general, ALDHs play significant roles in various cellular processes such as metabolism, oxidative stress response, detoxification of aldehydes, and cellular differentiation [11,12]. ALDHs constitute one of the aldehyde-metabolizing superfamilies [11] and are well-known metabolizers of pharmaceuticals, alcohol, and other pollutants. Mechanistically, ALDHs function as a family of nicotinamide adenine dinucleotide (phosphate) (NAD(P)^+^)-dependent enzymes that oxidize the NAD(P)^+^ of highly reactive exogenous and endogenous aldehydes to form carboxylic acids [13,14].

### 1.2. ALDH Isoenzymes and Their Correlations with Prognoses in Different Cancers

ALDH is a promising anti-cancer target due to its significant functional roles and abnormal expression in different types of cancers. For instance, in cancer types with low ALDH expression, conventional chemo- and radiation therapies are effective. However, high ALDH expression confers resistance to such therapies, and strategies involving inhibition of ALDH in combination with conventional therapies are promising [15]. ALDH is usually overexpressed in cancer, and each isoform has a unique expression pattern and distinct role in different cancer types. Immuno-staining normal SCs and cancer SCs (CSCs) for ALDH is usually positive and tracks the overpopulation of CSCs during colon tumorigenesis [3]. In clinical oncology, abnormal expressions of ALDHs are often correlated with patient prognosis (Table 1).

The 11 ALDH families are comprised of 19 ALDH isoforms: ALDH1A1, ALDH1A2, ALDH1A3/ALDH6, ALDH1B1/ALDH5, ALDH1L1/FDH, ALDH1L2/mtFDH, ALDH2, ALDH3A1, ALDH3A2/FALDH, ALDH3B1/ALDH7, ALDH3B2/ALDH8, ALDH4A1/P5CD, ALDH5A1/SSADH, ALDH6A1/MMSDH, ALDH7A1/EPD, ALDH8A1, ALDH9A1/ALDH4, ALDH16A1, and ALDH18A1/P5CS [15]. Among the 19 ALDH isoforms, the ALDH1 family/group is the most extensively studied due to their crucial involvement in both embryonic and adult SC maintenance and renewal processes [10]. Therefore, the rest of this review will focus on the ALDH1 family and their function in different cancers.

## 2. The ALDH1 Family

### 2.1. ALDH1 Structure

In humans, ALDH1 is primarily located in the liver. A 53-kb ALDH1 gene is situated on chromosome 9 and encodes approximately 501 amino acids [16]. The structure of human ALDHs is highly conserved as a homomultimer with three structural domains on each monomer: a catalytic domain, an NAD(P) binding domain, and an oligomerization domain [17,18]. At the junction of these domains, there is a funnel-shaped cavity with an opening towards the catalytic pocket [19]. Inside this pocket is the ALDH1 active site—a vital catalytic thiol characterized by a cysteine residue [19,20]. The active site is required for catalysis initiation, where the aldehyde first interacts with a cysteine residue to form the tetrahedral intermediate [14,21]. Differences among ALDH enzymes are present in the channel used for the substrate’s entry into the active site, known as the substrate entry channel (SEC). The SEC contains three key amino acid (AA) residues responsible for substrate specificity: the “mouth” (AA124), which performs aldehyde size selection; the “neck” (AA459), located at the proximal third of the channel; and the “bottom” (AA303), situated at the end of the channel directly next to the cysteine residue in the catalytic pocket [22,23].

There are six isoforms that belong to the ALDH1 family: ALDH1A1, ALDH1A2, ALDH1A3, ALDH1B1, ALDH1L1, and ALDH1L2. However, ALHD1B1 and ALDH1L2 are mitochondrial enzymes; the rest of them are cytosolic [24]. They are also expressed in several other tissues (Table 2) (*The Human Protein Atlas*) with distinct biological functions. While the three isoenzymes—ALDH1A1, ALDH1A2, and ALDH1A3—are the critical proteins that participate in the RA pathway to regulate cellular proliferation and differentiation [25], ALDH1B1 mainly involved in acetaldehyde regulation and lipid metabolism [26], and ALDH1L1 and ALDH1L2 function in metabolizing folate [26]. Due to the involvement in multiple biological processes, the ALDH1 family is found to participate in oncogenic pathways and cancer progression. In fact, multiple ALDH1 genetic alterations in different cancers have been reported, which implies the significance of ALDH1 in cancer development (Table 2).

### 2.2. Functions of ALDH1 in Normal Tissues

**Figure 2:**
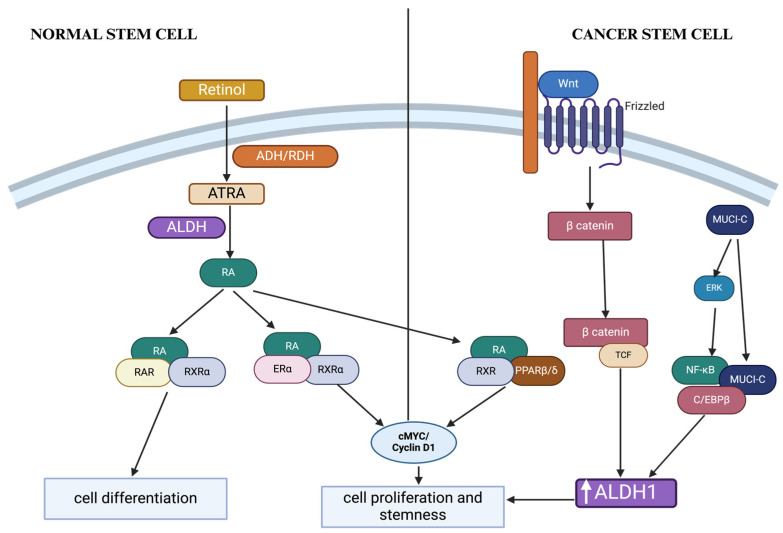
ALDH1 functions in normal and cancer stem cells. ALD = alcohol dehydrogenase; ALDH = aldehyde dehydrogenase; ATRA = all-trans-retinoic acid; ERα = estrogen receptor alpha; PPARβ/δ = peroxisome proliferator-activated receptors beta/delta; RA = retinoic acid; RARα = retinoic acid receptor alpha; RARβ = retinoic acid receptor beta; RDH = retinol dehydrogenase; ROS = reactive oxygen species; RXR = X retinoid receptor; TCF = T-cell factor. ALDH plays a role in the RA pathway as it converts ATRA to RA. In normal stem cells, RA binds to the receptors to form complexes RA/RAR/RARα to trigger cellular differentiation or RA/Erα/RXRα to trigger cellular proliferation and enhance stemness. In cancer stem cells, oncogenic pathways, such as *WNT* signaling and MUC1-C, upregulate *ALDH1* transcription. *ALDH1* is suggested to reduce oxidative stress, thus protecting the CSC population.

ALDH1 is an essential enzyme involved in retinoid metabolism. Absorbed retinol (vitamin A) is oxidized by the retinol dehydrogenase enzymes to retinal [47]. The ALDH1 family, including ALDH1A1, ALDH1A2, and ALDH1A3, are actively involved in retinoid metabolism by catalyzing retinal to RA [11]. RA is critical for embryological development, gene regulation, and morphogenesis [48]. In the traditional pathway, RA products such as 9-cis-retinoic acid (9-cis-RA), 13-cis-retinoic acid (13-cis-RA), and ATRA bind to RARA before entering the nucleus to promote transcription of c-MYC and cyclin D1. Alternately, RAs can bind to RARA and retinoid X receptor (RXR) dimers to induce RARB, resulting in cell differentiation (Figure 2) [48,49]. ALDH1A1 also plays a role in the regulation of RA signaling as ALDH1A1 levels might be controlled by intracellular RA in order to increase RA production in circumstances when the endogenous RA concentration becomes low [11,47].

Additionally, ALDH1 plays an important role in the oxidative-defense mechanism by catalyzing aldehydes. Reactive aldehydes, generated from alcohols, neurotransmitters, and environmental pollutants, can cause DNA damage, alter signaling pathways, and contribute to carcinogenesis [47]. ALDH1A1, along with ALDH2, catalyzes aldehydes resulting from alcohol metabolism to reduce oxidative stress. These reactive aldehydes include, among others, substrates like 4-hydroxy-2-nonenal (4-HNE) and malondialdehyde (MDA), which are processed by ALDH1B1. Apart from mitigating alcohol-induced oxidative stress, ALDHs (specifically ALDH1A1 and ALDH3A1) play a role in neutralizing reactive aldehydes generated by UV radiation, preventing the formation of 4-HNE and MDA [47].

### 2.3. Functions of ALDH1 in Normal Stem Cells

Previous studies have consistently shown the presence of ALDH expression in the SCs of various tissues, indicating its association with stemness [50]. Reportedly, ALDH1 activity is predominantly high in hematopoietic progenitors, neural SCs, and adipose SCs [51]. Additionally, ALDH1 is proposed to be involved in stem cell functions, such as self- protection, differentiation, and expansion [47]. ALDH1 also protects cells from many cytotoxic drugs, such as cyclophosphamide and 4-hydroperoxycyclophosphamide (4-HC) [11]. According to in vitro experiments by Singh et al. and Ma et al., ALDH1 inhibition leads to many hematopoietic SCs (HSCs) being stuck in the G0 phase [52,53].

Since ALDH1 activity in normal SCs is tissue-specific, Deng and colleagues categorized tissues into three groups based on the degree of ALDH1 expression: (1) tissues exhibiting either no expression or limited expression, exemplified by lung and breast tissues, (2) tissues demonstrating comparatively weaker expression in contrast to others, such as gastric epithelium and colon, and (3) tissues displaying a higher level of expression, including the liver and pancreas [54].

## 3. ALDH1 in Cancers

Upregulation of *ALDH1* has been reported in several types of cancer and is often associated with poor prognosis and a high rate of treatment resistance. Thus, *ALDH1* has become an “attractive” target in cancer therapies. Several approaches have been developed to inhibit *ALDH1* in an attempt to improve cancer patients’ outcomes. A summary of current *ALDH1* inhibitors is presented in Table 3.

### 3.1. Functions of ALDH1 in CSCs

Besides being a marker in normal SCs, ALDH1 is also considered a marker of CSCs due to its association with stemness. Previous studies have observed a higher expression level of ALDH1 in tumor tissues compared to normal tissues [55,56]. ALDH1 is proposed to have the ability to maintain CSC characteristics through RA metabolism [11]. In the non-traditional pathway, RA can activate the phosphoinositide 3-kinase (PI3K) signaling pathway and reduce the activity of protein kinase C to induce apoptosis inhibition and proliferation [57]. Moreover, RA can form heterodimers with estrogen receptor alpha (ERα) and peroxisome proliferator-activated receptors beta/delta (PPARB, PPARD) to promote cell survival (Figure 2) [48,53].

ALDH1 also plays a critical role in cell protection by reducing oxidative stress [48] in CSCs. Reportedly, ALDH1 transcription is upregulated by oncogenic pathways, such as MUC1C, ERK, Notch-DLL4, and WNT/β-catenin (Figure 2) [57].

### 3.2. ALDH1 in Breast Cancer

Breast cancer is a major public health concern and appears to be the most common cancer and the second-leading cancer-related cause of death among women worldwide [58]. Despite recent advances in the understanding of breast cancer markers and mechanisms, which have greatly improved the survival rate of patients, it remains incurable due to the inability to identify and target CSCs [56]. The invasion and metastasis of breast cancer involve specific subsets of tumor cells that express SC-like characteristics, with ALDH1 being one of the markers associated with these features [59]. Therefore, along with its role in retinoid metabolism, ALDH1 becomes a prominent marker to distinguish between normal SCs and CSCs in breast tissue [60,61].

Among the members of the ALDH1 family, ALDH1A1 and ALDH1A3 have a much more dominant role in upregulating ALDH1 activity, potentially resulting in a poor prognosis in breast cancer [62]. ALDH1A1 upregulates granulocyte–macrophage colony-stimulating factor (GMCSF) by activating the TAK1-NFkB signaling pathway, leading to myeloid-derived suppressor cells (MDSCs) expansion and resulting in decreased immune responses toward breast tumors [63]. Additionally, ALDH1A1 can maintain local cellular pH by up-regulating USP228/MYC signaling to promote breast CSCs [64]. Moreover, ALDH1A1, the breast cancer marker for stemness, initiates the ALDH1A1/HIF-1α/VEGF pathway through RA signaling. The activation of HIF-1α induces the expression and release of VEGF, thereby promoting tumor angiogenesis [65]. Several studies have found that the activation and transcription of ALDH1 are primarily associated with several pathways including MUC1-C/TWIST1/EMT [66], MUC1-C/ERK/CEBPβ/ALDH1A1 [67], Nanog [68], WNT/β-catenin [69], Notch and TGF-β [70], the SIRT1-PRRX1-KLF4-ALDH1 [71], IL-6/STAT3/ALDH1 [72], and other related pathways that contribute to CSC progression and metastasis.

In breast cancer, elevated ALDH1 expressions, particularly ALDH1A1 and ALDH1A3, are associated with chemoresistance, particularly cyclophosphamide-based regimens [56,73]. Inhibiting these two isoforms resulted in reverse chemoresistance in breast cancer. In 2016, Kida et al. analyzed 234 breast cancer patients who were treated with neoadjuvant chemotherapy, and they found that in ALDH1(+) cases, the pathological complete response (pCR) was significantly lower (13.5% vs. 30.3%) [74]. Moreover, knocking down these two isoenzymes, ALDH1A1 and ALDH1A3, resulted in decreased ALDH activity, leading to decreased therapy resistance and metastatic behavior in breast cancer cells [75].

Due to the significant involvement of ALDH1 in breast CSCs, recent breast cancer therapies exploit ALDH1 as a target. One promising approach is the cold atmospheric plasma (CAP) targeting ALDH1 breast CSCs through the ubiquitination of AQP3-5K and FOXO1 K48 mediated by AQP3-19Y [76], which yields a therapeutic effect. Tumor cell viability is reduced with the combination of radiation therapy, paclitaxel therapy, and N,N-diethylaminobenzaldehyde (DEAB), which targets ALDH1 in breast CSCs [68]. Moreover, breast CSC growth can be inhibited by ALDH1A1 inhibitors, such as limonin [77], quercetin [78], and Disulfiram [63]. ALDH1-targeted therapies have yielded promising outcomes, although more studies and trials are necessary to optimize the effect.

### 3.3. ALDH1 in Colorectal Cancer

CRC is the second-leading cause of cancer-related deaths worldwide, following lung cancer [79]. Most deaths related to CRC are attributed to metastasis, chemoresistance, and recurrence resulting from CSCs within the tumor [80,81]. Like breast cancer, CRC tumor tissues exhibit abundant ALDH1 expression, making ALDH1 a marker for CRC CSCs [82]. Several studies have identified a role for ALDH1 in several oncogenic pathways implicated in CRC. ALDH1 likely plays a role in the WNT/β-catenin pathway, where ALDH1B1 helps protect CSCs from DNA damage [80,82,83]. ALDH1 promotes CSC growth via the PI3K/AKT/mTOR signaling pathway [71,81]. Upregulation of ALDH1 expression occurs with mutant p53 and P2X7R [84,85].

ALDH1 proteins, particularly ALDH1A1 and ALDH1A3, are implied to contribute to chemoresistance in CRC. ALDH1A3 is found to be a specific isoform that was overexpressed in chemoresistant derivatives CRC cells, along with alterations in the expression of CSC markers: CD133, CD166, CD24, CXCR4, CD26, CD271, and CD274 [86]. Moreover, knocking down ALDH1A1 or ALDH1A3 by siRNA was found to increase the cytotoxicity effects of capecitabine and 5-FU in CRC cell lines: HT-29/eGFP, HCT-116/eGFP [87].

Given that CSCs confer resistance to conventional therapies, inhibiting or eliminating colorectal CSCs is necessary to effectively treat CRC and prevent recurrence; thus, ALDH1 is an attractive target for potential CRC therapies. ALDH1A1 inhibition can reduce oxidative phosphorylation and downregulate the retinol metabolism pathway [83]. In 2019, Yang and colleagues conducted a study on the inhibitory effect of physisorption towards colorectal CSCs by inhibiting ALDH1 through the Sonic Hedgehog and Notch signaling pathways [88]. Additionally, ALDH1 can be downregulated through the E-Cadherin/β-Catenin pathway modulation by treating with silibinin or the Hh signaling pathway by treating with tumidulin [89,90]. Several studies have been conducted to investigate co-factors to indirectly inhibit ALDH1, which have found that downregulation of either DCLK1, NEAT1, or KDM2B [81,91,92] not only inhibits ALDH1 expression in CRC but also alleviates invasion and chemoresistance.

### 3.4. ALDH1 in Lung Cancer

Lung cancer (LC) is the deadliest cancer worldwide [58], mostly due to the phenomenon of treatment resistance. ALDH1 has been confirmed as a lung CSC marker [93], and its high expression is associated with a poor prognosis in LC patients [49]. Activation of the MEK/ERK pathway is found to upregulate ALDH1 expression, activating the RA signaling pathway in LC cells [94]. Moreover, SOX9, β-catenin, and STAT3 are all involved in pathways that regulate ALDH1 expression in LC cells [95,96,97]. However, the details of the mechanisms and pathways that ALDH1 employs to drive tumor cell expansion and resistance remain unclear.

In 2020, Rebollido-Rios et al. conducted a study that found ALDH1 can indirectly induce chemoresistance in non-small cell LC by promoting changes in the glutathione redox system [98]. Moreover, ALDH1A1 can facilitate lung adenocarcinoma cells to dramatically proliferate in response to drug stress through the Warburg effect [99].

Since ALDH1 overexpression is negatively associated with treatment outcomes in LC, several studies of ALDH1-targeting treatments have been conducted. Many of them reported that ALDH1 inhibition can reduce tumor growth and treatment resistance in LC, such as the S100A9-ALDH1A1-retinoic acid signaling pathway-targeting study that showed a reduction in recurrence in LC [94]. Additionally, Wei and colleagues discovered that eliminating ALDH1A1 would result in a significant increase in the rate of apoptosis and a decrease in drug resistance [56]. Numerous other studies have been conducted to target ALDH1, mainly through the WNT pathway, NF-κB pathway, and WNT/β-catenin/STAT3 axis, which have yielded promising results [100,101].

### 3.5. ALDH1 in Gastric Cancer

Gastric cancer (GC) is ranked as the fifth most common cancer type worldwide [58]. Due to limited treatment options, GC patients have a poor survival rate, likely resulting from chemoresistance. Studies conducted on GC revealed that ALDH1 expression in tumor tissue was significantly higher than in normal tissue, and the level of ALDH1 expression is correlated with the tumor stage, metastasis stage, and treatment outcomes [102,103]. In addition, recent results propose that the high level of ALDH1 expression is one factor that plays an important role in chemoresistance and metastasis [102]. Overexpressed ALDH1 cells in GC can interfere with macrophage function by antagonizing macrophage-secreted effector molecules, thus escaping apoptosis and promoting tumor growth and invasion [102]. Moreover, Song et al. discovered that upregulation of TAZ and inhibition of HMGA2 will enhance ALDH1 expression in tumor tissue in GC [104].

To address the challenges in the current treatment for GC, extensive research on new therapeutic targets has been conducted. ALDH1 has gained considerable attention from scientists due to its physiological function in gastric CSCs and from recent studies showing that inhibiting ALDH1 expression by ATRA can inhibit tumor growth and reverse chemoresistance in GC [105]. In 2022, Wang and colleagues discovered that targeting ALDH1A1 can inhibit tumor viability through the WNT pathway in the invasion of MKN-45 cells [106]. Additionally, they proposed several methods to target ALDH1A1 in GC, including siRNA, overexpression of RORβ, and salinomycin [106]. In another study conducted by Gong et al., miR-95 was implicated in ALDH1 regulation, and silencing miR-95 inhibits ALDH1 expression in GC cells [107]. These studies consistently propose that ALDH1 can be a promising target for gastric tumor suppression, and further investigations are necessary.

### 3.6. ALDH1 in Cervical Cancer

Cervical cancer is the fourth most common cancer in women globally and has a high recurrence rate (50–70%) in advanced stages [108]. Several studies have identified ALDH1, based on its overexpression, as a critical marker for cervical cancer SCs (CSCs), which drive chemotherapeutic resistance [109]. The ALDH1+ sub-population of cervical cancer cells exhibits high tumorigenicity, including dysregulated cellular proliferation and migration [109]. Consequently, ALDH1 is significantly correlated with poor prognosis in cervical cancer patients.

In cervical cancer, ALDH1 expression is regulated by various mechanisms, including the activation of Erk1/2 and AKT signaling pathways, which are triggered by the binding of miR-222 to the 3′ untranslated region of ALDH1 mRNA [110,111]. Previous studies have suggested that the upregulation of ALDH1 promotes other CSC biomarkers, such as Nanog, Sox2, Oct4, and Twist1. However, the mechanisms through which ALDH1 regulates these stemness transcription factors remain poorly understood. The most broadly accepted hypothesis is that ALDH1, particularly ALDH1A1, plays a crucial role in the RA pathway by activating the transcription of genes involved in SC differentiation [112]. Moreover, ALDH1 is found to be upregulated under hypoxic conditions, which are commonly observed in radioresistant cells. This upregulation helps these cells escape cell death by improving DNA damage responses [109].

In 2023, Fahmi and colleagues conducted a study to evaluate the correlation between ALDH1 expression and the risk of treatment resistance in cervical cancer. Their findings revealed a positive correlation between high ALDH1 expression (score ≥ 166.05) and an increased risk of incomplete response to radiation in stage III cervical cancer patients [109]. This result indicates that ALDH1 can serve as a predictive marker for both survival outcomes and the rate of treatment resistance. Given the biological significance of ALDH1 in cervical cancer, several studies have focused on developing therapeutic strategies targeting ALDH1. Promising inhibitors, such as compound 974, PM01183, Zoledronic acid, ATRA, ALDHi 673A, and Limonin, have shown effectiveness in inhibiting ALDH1 activity, thereby reducing tumor proliferation and stemness in cervical cancer [56,113,114]. Despite these promising findings, further research is needed to enhance our understanding of ALDH1-based mechanisms in regulating stemness in cancer cells.

### 3.7. ALDH1 in Melanoma

Melanoma, the deadliest form of skin cancer, is also one of the most common cancers among young adults. ALDH1 has been found to be overexpressed in melanoma, with ALDH1A1 and ALDH1A3 being the two predominantly expressed isoforms [115,116]. Along with its overexpression, ALDH1 has been identified as a key factor driving treatment resistance in melanoma through CSC upregulation [116]. In 2012, Luo and colleagues discovered that silencing the ALDH1A gene resulted in apoptosis due to cell cycle arrest in human melanoma cells, highlighting its crucial role in melanoma development [117].

Furthermore, ALDH1A3 appears to significantly influence treatment outcomes. In melanoma patients with BRAF mutations, high ALDH1A3 expression correlates with favorable responses to BRAF/MEK inhibitor therapy [118]. Conversely, low ALDH1A3 expression is associated with increased inflammatory responses, including upregulation of CD8+ T-cells, plasma cells, and macrophages [119]. These findings suggest that ALDH1A3 could be a key regulator of immune and treatment responses in melanoma.

Several studies also indicate that ALDH1 is a target gene of the WNT/β-catenin pathway, a critical signaling pathway in melanoma progression [120]. Additionally, ALDH1 is hypothesized to protect cancer cells from oxidative stress by detoxifying reactive oxygen species (ROS), thereby promoting melanoma cell survival [121]. Based on the accumulating evidence of ALDH1’s role in melanoma, multiple studies have investigated ALDH1 as a potential target for melanoma treatments. Several ALDH inhibitors, such as KS100, Nifuroxazide, and Disulfiram, have shown promising results in preclinical studies [122,123]. Collectively, ALDH1 remains a crucial diagnostic biomarker and a predictive factor for treatment response, making it a potential target for personalized therapies in melanoma.

### 3.8. ALDH1 in Prostate Cancer

Prostate cancer is the second-leading cause of cancer-related death among American men. Notably, ALDH1, particularly ALDH1A1, has been identified as a biomarker for CSCs in prostate cancer and is clinically associated with poor outcomes [124]. Studies have shown that ALDH1A1+ prostate cancer cells exhibit high tumorigenicity and can promote the progression of tumors transplanted in mice. These transplantable tumors histopathologically resemble the parental tumors, indicating the crucial role of ALDH1A1 in stemness regulation in prostate cancer [124].

Moreover, the distribution of ALDH1A1 in normal and tumor prostate tissues is quite distinct. In normal prostate tissues, ALDH1A1+ cells are primarily located in the basal component. In tumor tissues, however, ALDH1A1+ cells are detected in both secretory epithelial cells and NECs [124]. Recent research published in 2024 led to the discovery that ALDH1A1 promotes metastasis and treatment resistance in prostate cancer through interactions with RAR-dependent transcription and the androgen receptor (AR) [125]. These interactions increase as the tumor progresses, and ALDH1A1 notably induces tumor survival [125]. Additionally, the study revealed that ALDH1A1 overexpression is associated with high expression of Polo-like kinase 3 (PLK3) in prostate cancer bone metastases, suggesting that ALDH1A1 regulates PLK3 through its interplay with AR and RAR-dependent transcription. ALDH1A1-based regulation of PLK3 enhances cellular proliferation and migration, driving tumor progression and metastasis in prostate cancer [125]. This study, along with others, highlights the biological significance of ALDH1A1 in prostate cancer, positioning it as a promising therapeutic target to prevent metastasis and overcome treatment resistance.

### 3.9. ALDH1 in Kidney Cancer

Renal cancer is among the top ten most common cancers in the United States, with renal cell carcinoma (RCC) being the most common type. ALDH1, particularly ALDH1A1, is overexpressed and correlated with poor prognosis in RCC [126]. The overexpression of ALDH1A1 is reported in 56.8% of clear cell RCC (ccRCC) samples, and high ALDH1A1 expression is associated with tumor stage, invasion, and recurrence risk [126].

Nonetheless, findings on ALDH1 expression patterns in RCC can vary. While some studies indicate that elevated ALDH1 expression is linked to poor outcomes in RCC [126,127,128], others claim that ALDH1 expression is not significantly associated with cancer stage or tumor grade [129]. This discrepancy underscores the need for further research and alternative approaches to accurately characterize ALDH1’s role in RCC.

**Table 3:** Current ALDH1 inhibitors in cancer research.

Agent	Mode of Action	Cancer (s)	Preclinical Results	Ref.
4-diethylaminobenzaldehyde (DEAB)	Pan-ALDH inhibitor, with a K_i_ of 4 nM for ALDH1	Pancreatic cancer	DEAB weakens malignant proliferation of cancer cells, induces cancer cell apoptosis, and reduces gemcitabine resistance in pancreatic cancer cells.	[130]
673A	Pan-ALDH1 inhibitor (inhibited ALDH1A1 (IC_50_ 246 nM), ALDH1A2 (IC_50_ 230 nM), and ALDH1A3 (IC_50_ 348 nM) with minimal or no inhibition of ALDH2 (IC_50_ 14 μM) or numerous other ALDH family members).	Ovarian cancer	Reduces CD133^+^ cells in A2780 cell line with IC_50_ by approximately 10 uM. 673A triggers necroptosis in ovarian CSCs and induces expression of the mitochondrial uncoupling proteins. 673A is found to be effective in vivo.	[131]
ALDH1A1-IN-4	A potent inhibitor of ALDH1A1(IC_50_ = 0.32 μM).	Lung cancer Pancreatic cancer	Shows significant potency to reverse mafosfamide (an analogue of cyclophosphamide) resistance.	[132]
ALDH1A3-IN-1 (Compound **14**)	A potent inhibitor of ALDH1A3 (IC_50_ = 0.63 μM).	Prostate cancer	ALDH1A3-IN-1 reduces cell viability of primary prostate epithelial cultures in dose-dependent manner.	[133]
ALDH1A3-IN-3 (Compound **16**)	Potent inhibitor of ALDH1A3	Breast cancer	Combination of ALDH1A3-IN-3 and doxorubicin (DOX) significantly increased the inhibitory effect on cell viability in MCF7 cell line.	[134]
ATRA	ATRA can inhibit the growth of tumors derived from ALDH-High cells but not tumors derived from ALDH-Low cells.	Ovarian cancer NSCLC	Significantly reduces the increased expression of ALDH1A1 and CD44. Thus, it is suggested to enhance gefitinib-induced growth inhibition of NSCLC/ADC cells.	[135]
Citral	Selectively inhibits ALDH1A3	Breast cancer	Citral inhibits cellular proliferation, induces apoptosis and cell cycle arrest in human breast cancer cell line MCF-7.	[136]
CM-39	Reversible inhibitor for ALDH1A (IC_50_ = 0.9 μM).	Ovarian cancer	CM-39 displays synergistic effect with cisplatin and reduces cancer stem cell pool.	[137]
CM010	Potent and selective ALDH1A family inhibitor, with IC_50_s of 1700, 740, and 640 nM for ALDH1A1, ALDH1A2, and ALDH1A3, respectively	Ovarian cancer	CM10 inhibits ALDEFLUOR activity in live ovarian cancer cells and preferentially depletes CD133^+^ cells.	[131]
Compound 974	Selectively block ALDH1A1	Ovarian cancer	IC_50_ = 14.51 uM (OVCAR5) and 17.41 uM (OVCAR3) (48 h post-treatment) Compound **974** suppresses ovarian cancer stemness in vitro and in vivo.	[138]
Dimethyl ampal thiolester (DIMATE)	Inhibits ALDH1 and ALDH3	Leukemia	DIMATE prompts apoptogenic aldehyde accumulation, inducing apoptosis in leukemic cells but not toxic for healthy hematopoietic stem cells.	[139]
Disulfiram	Disulfiram competes with nicotinamide adenine dinucleotide (NAD) at the cysteine residue in the active site of ALDH1A1.	Non-small cell lung cancer (NSCLC)	Disulfiram significantly inhibits NSCLC stem cells and stem cell transcription factors Nanog, Sox2, and Oct-4 both in vitro and in vivo.	[140]
GA11	A potent ALDH1 inhibitor	Glioblastoma	GA11 displays antitumor effects in glioblastoma both in vitro and in vivo.	[141]
Gossypol	Gossypol binds to an allosteric site on ALDH1L1, a folate metabolism enzyme, and prevents NADP+ binding. This disrupts folate metabolism and reduces ATP production.	Breast cancer Pancreatic cancer Colorectal cancer Cervical cancer NSCLC	Inhibits cell viability in multiple cancer cell lines.	[142]
IGUANA-1	Potent inhibitor of ALDH1B1	Colorectal cancer	IGUANA-1 selectively blocks the growth of colon cancer spheroids and organoids.	[143]
KS106	A potent ALDH inhibitor with IC_50_s of 334, 2137, and 360 nM for ALDH1A1, ALDH2, and ALDH3A1, respectively.	Colorectal cancer Melanoma multiple myeloma	KS106 increases ROS activity, cell cycle arrest at G2/M phase, and apoptosis in multiple cancer cells. It reduces cell viability with IC_50s_ of 2.1–5.7 μM (melanoma), 2.5–5.8 μM (colorectal cancer), and 0.3–4.7 μM (multiple myeloma).	[144]
KS124 (Compound **3**)	KS124 is a potent inhibitor that inhibits ALDH1A1, ALDH1A3, ALDH3A1	Colorectal cancer	KS124 induces apoptosis and increases ROS production in HCT-116 cells (3 uM, 24–48 h).	[145]
NCT-505	Selective theophylline-based inhibitor that inhibits ALDH1A1	Ovarian cancer	Significantly reduces tumor-initiating cell viability at IC_50_ = 67.1 uM (OVCAR3) and 1.72 uM (OVCAR8) (*p* < 0.01). Reduces sphere formation capacity of ovarian cancer cells. Reduces carboplatin-treated viable cells after relapse in vitro.	[146]
NCT-506	Orally bioavailable inhibitor of ALDH1A1 (IC_50_ = 7 nM)	Ovarian cancer	NCT-506 inhibited the formation of 3D spheroid cultures of ovarian cancer cells, and it enhanced the cytotoxicity of paclitaxel in resistant ovarian cancer cell line, SKOV-3-TR.	[147]

## 4. Interactions of ALDH with Other Related Proteins or Pathways

The main protein–protein interaction of ALDHs involves its homo-dimerization. In ALDH’s formation of dimers and tetramers, binding takes place between the catalytic domain of one subunit and another subunit’s NAD(P)^+^ domain [13,148]. The homo-dimerization occurs through an oligomerization domain that is conserved among ALDHs. The catalytic domain is ALDH’s active site, where an aldehyde substrate binds to specific amino acid residues that facilitate the oxidation reaction. The NAD(P)^+^-binding domain in ALDH is where the NAD(P)^+^ cofactor binds [13,148] to facilitate the redox reaction catalyzed by ALDH. Thus, the formation of a stable dimer or tetramer leads to a functional enzyme that catalyzes the oxidation of aldehydes to carboxylic acids.

The ALDH enzymes are crucial to alcohol metabolism, particularly ALDH2. However, mutations in ALDH that disrupt its interaction with NAD+ can lead to adverse reactions to alcohol [149,150]. The mechanism by which ALDH variants affect alcohol intolerance involves the elevation of acetaldehyde levels resulting from slower acetaldehyde oxidation [150,151]. Acetaldehyde causes a highly aversive reaction, including facial flushing, nausea, and tachycardia. Indeed, the ALDH2*2 variant with a single nucleotide polymorphism E487K that resides in the oligomerization domain is associated with cardiovascular disease, cancer, alcohol intolerance, and Alzheimer’s disease [13].

Several other protein interactions can occur that depend on the ALDH isoform and sub-cellular location. Some interactions involve aldehyde metabolism through the binding of ALDH with other metabolic enzymes. Additional interactions can occur with regulatory proteins such as transcription factors and kinase enzymes. Some of these interactions are ALDH isoform-specific. For example, ALDH16A1 can interact with hypoxanthine-guanine phosphoribosyltransferase (HPRT1), which affects uric acid metabolism [152]. Certainly, ALDH16A1 is a non-catalytic enzyme that appears to play a role in the etiology of gout through its interaction with HPRT.

Also, ALDH2 can interact with mitochondrial proteins involved in the detoxification of acetaldehyde and endogenous lipid aldehydes, suggesting a protective role against heart disease [153]. Indeed, many (~40%) East Asians who carry a single nucleotide polymorphism (ALDH2 rs671) have an increased risk of cardiovascular disease (CVD). ALDH2 rs671 mutant attenuates the interaction of low-density lipid receptor (LDLR) and ALDH2, which provides a molecular mechanism by which ALDH2 rs671 SNP plays a role in atherosclerosis and CVD [154].

Another example relates to ALDH’s role in retinoid signaling involving the generation of RA, which activates retinoid receptors (RARs and RXRs) and influences gene expression. However, ALDH1A1 can interact with the protein arginine methyltransferase 3 (PRMT3), regulate ALDH activity by methylation, and inhibit retinoid signaling [155]. Moreover, ALDH activity can be influenced by post-translational modification, including acetylation, phosphorylation, and nitration [156]. Thus, a host of ALDH interactions with other proteins can impact different cellular functions and pathways as well as disease processes.

## 5. Impact of ALDH on the Tumor Microenvironment and Its Interaction with Immune Cells

ALDH has a significant role in the tumor microenvironment, particularly the impairment of immune response [157]. Two key points bear further discussion:

1. Resistance to chemotherapy. ALDHs are often overexpressed in stem cells, which display resistance to systemic therapies. In cancer stem cells, chemotherapy resistance occurs through multidrug efflux pumps, detoxification of toxic aldehydes generated from chemotherapy, prevention of reactive oxygen species formation, as well as the decrease in oxidative stress, DNA damage, and apoptosis [15,158]. Indeed, the enzymatic activity of ALDH has a direct role in drug resistance via the detoxification of endogenous and exogenous aldehyde substrates via NAD(P)^+^-dependent oxidation. ALDH’s ability to detoxify aldehydes is dependent on the substrate specificity of different ALDH isoforms [159,160,161]. The classic example is the activity of ALDH1A1 and ALDH3A1 which function as a major mediator of resistance to the alkylating agent cyclophosphamide [162].

Accordingly, targeting aldehyde dehydrogenase enzymes in combination with chemotherapy and immunotherapy offers a new approach to overcoming drug resistance in oncology. While various ALDH inhibitors are available [163], N,N-diethylaminobenzaldehyde (DEAB) is the main inhibitor that has been investigated against human ALDH isoenzymes. Recent enzyme kinetic analyses and QTOF mass spectrometry studies show that DEAB is a substrate for many ALDH isoenzymes, especially ALDH3A1. Notably, its electronic features generate a stalled acyl-enzyme ALDH intermediate that stabilizes the electronic resonance structure [164]. This renders DEAB a mechanism-based irreversible inhibitor for ALDH2 and ALDH1A2, a very slow substrate-type inhibitor for ALDH1A3, ALDH1B1, and ALDH5A1, and a slow substrate-type inhibitor for ALDH1A1. While translating ALDH inhibitors into the clinic may theoretically be an important approach to overcome therapy resistance, challenges still exist. For example, inhibiting too many ALDH family members could be toxic, and isoform-specific inhibition may not be possible. Thus, the promise of anti-ALDH agents in cancer therapeutics has yet to be realized.

2. Evasion of immune response. Several studies reveal that not only is ALDH overexpression important for the survival of cancer stem cells but also that ALDH-mediated metabolism of aldehydes can promote evasion of an immune response. For example, Terzuoli et al. showed that ALDH can influence Programmed Death-Ligand 1 (PD-L1) expression in tumor cells [165]. Specifically, they found ALDH3A1 overexpression enhanced PD-L1 output in tumor cells, and ALDH3A1 expression correlated with PD-L1 expression in melanoma and lung cancer patient specimens. Jancewicz et al. also showed that breast cancer cells expressing PD-L1 can attenuate human effector CD4+ T cells manifesting high PD-1 and PD-L1 expression levels [166]. Thus, overexpression of ALDH1 and increased PD-L1 levels appear to contribute to tumor evasion of immune response by inhibiting T-cell activity. Additionally, Eichberger et al. reported that modulating PD-L1 levels in head and neck cancer cells influenced cell spreading, migration, and invasion [167]. Moreover, López et al. found that ALDH1A1 levels correlated with PD-L1 and tumor-infiltrating lymphocytes in breast cancer patients with pathologic response and improved survival [168]. Other studies by Wang et al. demonstrated that ALDH1A1 promotes immune escape of tumor cells through the ZBTB7B-glycolysis pathway [169]. In a further study, Guo et al. reported that upregulation of PD-L1 expression by aerobic glycolysis promotes tumor immune evasion by hexokinase2-mediated phosphorylation of IκBα [170]. Finally, Liu et al. observed that ALDH1A1 decreased intracellular pH in breast cancer cells, activated NFκB signaling, and increased secretion of GM-CSF, which led to myeloid-derived suppressor cell expansion and immunosuppression [63]. Taken together, these later studies suggest that targeting ALDH1A1 and glycolysis in combination with immune checkpoint inhibitors could synergistically inhibit tumors in vivo.

## 6. Conclusions

In conclusion, ALDH1 plays a crucial role in cellular physiology across both normal and cancerous tissues. Recent studies have consistently highlighted the importance of ALDH1 in various cancers. Its expression has been linked to tumor stage and the risk of metastasis, positioning it as a potential prognostic marker and a promising therapeutic target.

To fully harness the potential of ALDH1 in cancer diagnosis and treatment, future research should focus on three key areas. First, there is a need to optimize potent and selective ALDH1 inhibitors. Given the diversity of isoforms and their distinct expression patterns across different cancer types, developing specific inhibitors for ALDH1 is essential for targeted therapies. Second, exploring combination treatments that include ALDH1-targeting strategies could be beneficial. Considering the genetic and phenotypic heterogeneity in cancers, combination therapies could simultaneously target multiple pathways, potentially leading to more favorable outcomes. Third, research should delve into the tissue-specific and cancer-specific expressions and functions of ALDH isoforms, particularly ALDH1. This could provide deeper insights into how ALDH1 regulates CSCs and influences tumor progression.

While our understanding of ALDH1’s structure and function, especially in the context of cancer, has significantly advanced over the past five years, challenges remain in fully elucidating its role in tumorigenesis. The complex interactions of ALDH1 with other key factors in the tumor microenvironment, along with its varied contributions across different cancers, necessitate further investigation. Bridging these knowledge gaps could lead to the discovery of new, innovative, and effective cancer treatments.

## Figures and Tables

**Table 1:** Human aldehyde dehydrogenase isoenzymes expression and cancers.

ALDH Isoenzyme	Cancer	Survival Analysis
ALDH1A1	Breast	5-year survival high expression: 85% 5-year survival low expression: 77% *p*-score: 0.0085 ***
	Colorectal	5-year survival high expression: 56% 5-year survival low expression: 66% *p*-score: 0.056
	Prostate	5-year survival high expression: 96% 5-year survival low expression: 98% *p*-score: 0.37
	Pancreatic	5-year survival high expression: 31% 5-year survival low expression: 26% *p*-score: 0.045 ***
ALDH1A2	Cervical	5-year survival high expression: 72% 5-year survival low expression: 60% *p*-score: 0.025 ***
	Colorectal	5-year survival high expression: 74% 5-year survival low expression: 58% *p*-score: 0.2
	Melanoma	3-year survival high expression: 29% 3-year survival low expression: 47% *p*-score: 0.009 ***
ALDH1A3/ALDH6	Renal	5-year survival high expression: 66% 5-year survival low expression: 74% *p*-score: 0.012 ***
	Colorectal	5-year survival high expression: 58% 5-year survival low expression: 63% *p*-score: 0.0062 ***
	Melanoma	3-year survival high expression: 47% 3-year survival low expression: 37% *p*-score: 0.096
	Ovarian	5-year survival high expression: 27% 5-year survival low expression: 34% *p*-score: 0.045 ***
ALDH1B1/ALDH5	Colorectal	5-year survival high expression: 63% 5-year survival low expression: 54% *p*-score: 0.058
	Lung	5-year survival high expression: 43% 5-year survival low expression: 50% *p*-score: 0.027 ***
	Liver	5-year survival high expression: 62% 5-year survival low expression: 42% *p*-score: 0.0028 ***
	Ovarian	5-year survival high expression: 24% 5-year survival low expression: 34% *p*-score: 0.13
ALDH1L1/FDH	Renal	5-year survival high expression: 73% 5-year survival low expression: 52% *p*-score: 2.4 × 10^−8^ ***
	Colorectal	5-year survival high expression: 67% 5-year survival low expression: 46% *p*-score: 0.091
	Pancreatic	5-year survival high expression: 11% 5-year survival low expression: 35% *p*-score: 0.0031 ***
	Thyroid	5-year survival high expression: 83% 5-year survival low expression: 96% *p*-score: 0.007 ***
ALDH1L2/mtFDH	Renal	5-year survival high expression: 53% 5-year survival low expression: 73% *p*-score: 8.2 × 10^−8^ ***
	Stomach	5-year survival high expression: 30% 5-year survival low expression: 49% *p*-score: 0.0032 ***
	Breast	5-year survival high expression: 75% 5-year survival low expression: 84% *p*-score: 0.06
ALDH2	Lung	5-year survival high expression: 47% 5-year survival low expression: 44% *p*-score: 0.0056 ***
	Pancreatic	5-year survival high expression: 15% 5-year survival low expression: 43% *p*-score: 0.082
	Colorectal	5-year survival high expression: 64% 5-year survival low expression: 53% *p*-score: 0.0041 ***
	Breast	5-year survival high expression: 84% 5-year survival low expression: 74% *p*-score: 0.0092 ***
ALDH3A1	Thyroid	5-year survival high expression: 94% 5-year survival low expression: 87% *p*-score: 0.029 ***
	Colorectal	5-year survival high expression: 54% 5-year survival low expression: 63% *p*-score: 0.11
	Pancreatic	5-year survival high expression: 13% 5-year survival low expression: 50% *p*-score: 0.0027 ***
	Breast	5-year survival high expression: 85% 5-year survival low expression: 72% *p*-score: 0.000031 ***
ALDH3A2/FALDH	Thyroid	5-year survival high expression: 96% 5-year survival low expression: 86% *p*-score: 0.014 ***
ALDH3B1/ALDH7	Colorectal	5-year survival high expression: 49% 5-year survival low expression: 67% *p*-score: 0.00012 ***
	Liver	5-year survival high expression: 39% 5-year survival low expression: 53% *p*-score: 0.0079 ***
	Pancreatic	5-year survival high expression: 25% 5-year survival low expression: 38% *p*-score: 0.00061 ***
ALDH3B2/ALDH8	Stomach	5-year survival high expression: 42% 5-year survival low expression: 26% *p*-score: 0.026 ***
	Thyroid	5-year survival high expression: 95% 5-year survival low expression: 84% *p*-score: 0.012 ***
	Renal	5-year survival high expression: 66% 5-year survival low expression: 75% *p*-score: 0.014 ***
ALDH4A1/P5CD	Breast	5-year survival high expression: 76% 5-year survival low expression: 83% *p*-score: 0.12
	Colorectal	5-year survival high expression: 72% 5-year survival low expression: 58% *p*-score: 0.014 ***
	Renal	5-year survival high expression: 70% 5-year survival low expression: 63% *p*-score: 0.0043 ***
	Cervical	5-year survival high expression: 75% 5-year survival low expression: 49% *p*-score: 0.00051 ***
ALDH5A1/SSADH	Melanoma	3-year survival high expression: 28% 3-year survival low expression: 63% *p*-score: 0.022 ***
ALDH6A1/MMSDH	Lung	5-year survival high expression: 47% 5-year survival low expression: 43% *p*-score: 0.00042 ***
ALDH7A1/EPD	Thyroid	5-year survival high expression: 84% 5-year survival low expression: 95% *p*-score: 0.0031 ***
	Liver	5-year survival high expression: 60% 5-year survival low expression: 37% *p*-score: 0.0016 ***
	Colorectal	5-year survival high expression: 66% 5-year survival low expression: 44% *p*-score: 0.021 ***
ALDH8A1	Liver	5-year survival high expression: 60% 5-year survival low expression: 44% *p*-score: 0.0043 ***
ALDH9A1/ALDH4	Pancreatic	5-year survival high expression: 33% 5-year survival low expression: 0% *p*-score: 0.00046 ***
	Colorectal	5-year survival high expression: 69% 5-year survival low expression: 55% *p*-score: 0.042 ***
ALDH16A1	Stomach	5-year survival high expression: 53% 5-year survival low expression: 31% *p*-score: 0.029 ***
	Renal	5-year survival high expression: 62% 5-year survival low expression: 74% *p*-score: 0.0019 ***
ALDH18A1/P5CS	Melanoma	3-year survival high expression: 84% 3-year survival low expression: 29% *p*-score: 0.041 ***

Data were derived from Human Protein Atlas database proteinatlas.org. ***: statistically significant correlation between high/low expression with 5-year/3-year survival. *p*-score: Log-rank *p*-value for Kaplan-Meier plot showing results from analysis of correlation between mRNA expression level and patient survival. Patients were divided based on level of expression into one of the two groups “low” (under cut off) or “high” (over cut off). The cut off is specific for each type of isoform/cancer.

**Table 2:** The ALDH1 family.

Isoenzyme	Chromosomal Location	Tissue Distribution	Preferred Substrate	Cancers Associated with Genetic Alterations in ALDH1	Ref.
*ALDH1A1*	9q21.13	Liver ***, testis, small intestine, stomach, pancreas, kidney, colon, gallbladder	Retinal	Breast cancer Colorectal cancer Pancreatic cancer Renal cancer Gastric cancer Melanoma Ovarian cancer Prostate cancer	[27,28,29,30,31,32,33,34,35,36]
*ALDH1A2*	15q21.3	Fallopian tube ***, ovary, testis, heart muscle, gallbladder, prostate, kidney, lung, breast	Retinal	Breast cancer Colorectal cancer Pancreatic cancer Renal cancer Oligodendroglioma Head and neck squamous cell carcinoma Medulloblastoma	[27,29,31,35,37,38]
*ALDH1A3*	15q26.3	Prostate ***, urinary bladder, testis, breast, esophagus, small intestine, colon, pancreas	Retinal	Breast cancer Colorectal cancer Pancreatic cancer Renal cancer Glioblastoma Gastric cancer Chronic lymphocytic leukemia Medulloblastoma	[27,28,29,31,32,35,39]
*ALDH1B1*	9p13.1	Liver ***, colon, kidney, prostate, esophagus, stomach, small intestine	Acetaldehyde	Breast cancer Colorectal cancer Glioblastoma Prostate cancer	[27,28,36]
*ALDH1L1*	3q21.3	Liver ***, kidney, cerebral cortex, ovary, breast	10-formyltetrahydrofolate	Esophageal adenocarcinoma Colorectal cancer Lung cancer Breast cancer	[40,41,42,43]
*ALDH1L2*	12q23.3	Pancreas ***, prostate, bladder, stomach, gallbladder.	10-formyltetrahydrofolate	Large B-cell lymphoma Clear-cell renal cell carcinoma Bladder cancer Colorectal cancer Lung cancer Breast cancer	[27,44,45,46]

The data were derived from The Human Protein Atlas Database proteinatlas.org. *** indicates the tissue that has the highest tau specificity score. Tau specificity score is a numerical indicator of the specificity of the gene expression across cells or tissues. The value ranges from 0 and 1, where 0 indicates identical expression across all cells/tissue types, while 1 indicates expression in a single cell/tissue type.

## Abbreviations

ALDH	aldehyde dehydrogenase;
RA	retinoic acid;
SC	stem cells;
CSC	cancer SCs;
CRC	colorectal cancer;
BC	breast cancer;
LC	lung cancer;
GC	gastric cancer;
NEC	neuroendocrine cell
